# *QuickStats*: Death Rates* Attributed to Excessive Cold or Hypothermia,^†^ by Urbanization Level^§^ and Sex — National Vital Statistics System, 2018–2020

**DOI:** 10.15585/mmwr.mm7107a6

**Published:** 2022-02-18

**Figure:**
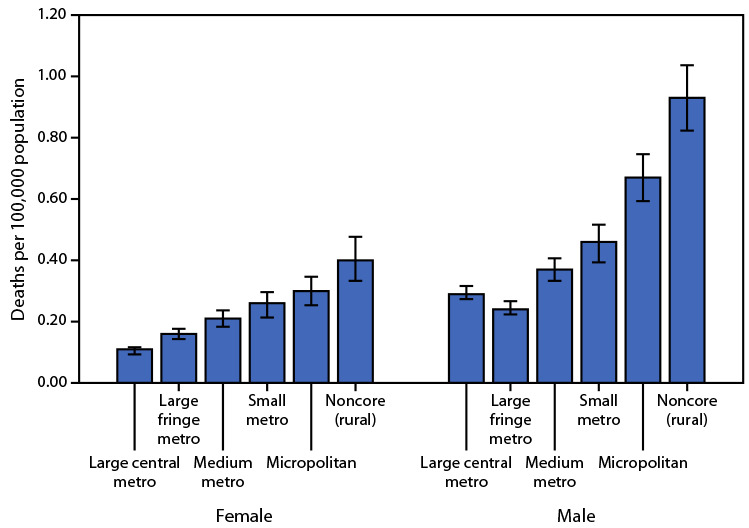
During 2018–2020, death rates attributed to excessive cold or hypothermia were generally higher in more rural areas. Among females, the death rate increased from 0.11 per 100,000 for those residing in large central metro areas, to 0.40 for those in noncore (rural) areas. Among males, the death rates were lowest for those residing in large central metro areas (0.29) and large fringe metro areas (0.24), and highest in noncore (rural) areas (0.93). Males had higher death rates than females for each corresponding urbanization level.

**Source:** National Center for Health Statistics, National Vital Statistics System, Mortality Data, 2018–2020. https://www.cdc.gov/nchs/nvss/deaths.htm

